# Ploidy‐Dependent Response to Anticancer Drugs of Human Embryonic Stem Cells

**DOI:** 10.1111/cpr.70278

**Published:** 2026-09-01

**Authors:** Guy Haim‐Abadi, Nissim Benvenisty

**Affiliations:** ^1^ The Azrieli Center for Stem Cells and Genetic Research, Department of Genetics, Silberman Institute of Life Sciences The Hebrew University Jerusalem Israel

**Keywords:** chemotherapy, human embryonic stem cells, ploidy

## Abstract

Polyploidy is normally found in numerous tissues and cell types in the generally diploid human body and is crucial for proper development. However, polyploidy, and especially triploidy, can also be found in malignant tumours and is often associated with chemoresistance, stemness and metastatic capabilities. Here, we utilise our isogenic haploid, diploid and triploid human embryonic stem cell (hESC) lines to study the effect of ploidy on the response to four different anticancer drugs. Surprisingly, we show that triploid cells are more sensitive to chemotherapy‐induced apoptosis and cell cycle arrest than diploid and haploid cells, correlated with higher levels of DNA damage. This phenotype is regulated by p53, as it was reversed in *TP53*‐KO cells, where triploid mutant cells display higher resistance to the chemotherapies we applied compared to mutant haploid and diploid cells. The reversal from sensitivity to resistance, driven by *TP53*‐KO of the triploid cells, was accompanied by a reversal in the enrichment of DNA repair, replication and cell division related genes, compared to their diploid counterparts. Conversely, triploidy triggered DNA damage‐induced differentiation in both WT and *TP53*‐KO treated cells, pointing to a ploidy‐dependent response not mediated by p53. In addition, we show that cancer cell lines also display similar ploidy‐dependent anticancer drug responses to our *TP53*‐KO hESCs. These findings uncover the interplay between ploidy and the p53 pathway in determining the outcome of anticancer therapeutics, and display the potential use of isogenic hESCs, differing only in their ploidy level, in studying the impact of ploidy on chemotherapy response.

## Graphical abstract

Using isogenic hESCs, differing only in their ploidy level, we show the effect of ploidy on the resistance to anticancer drugs. We demonstrate that polyploidy, by itself, confers sensitivity to chemotherapies, leading to higher apoptosis and delayed proliferation, while the loss of p53 reverses the phenotype, demonstrating higher resistance.

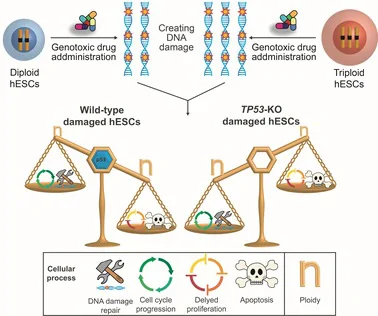

## 1 Introduction

The human body is comprised of mostly diploid cells, but some tissues and cell types vary from this state of ploidy. Polyploidy, that is, the presence of more than two copies of the genome, can be naturally found in several tissues and cell types in the human body, such as skeletal myocytes, hepatocytes and megakaryocytes [1, 2, 3, 4]. The only haploid cells in our body are the germ cells; however, haploid human embryonic stem cells (hESCs) were isolated by us via the activation of unfertilized human oocytes [5]. These cells were isolated from the inner cell mass of the derived blastocysts and then propagated in vitro. These cells were subsequently characterised as pluripotent stem cells and were shown to function and differentiate properly, much like diploid cells [5]. Similarly, we recently generated isogenic triploid hESC lines and demonstrated the major similarity between these cells and diploid cells, including their transcription, methylation and DNA replication patterns [6].

Polyploidy is often observed in malignant tumours, where it is linked to the acquisition of cancerous features like tumorigenicity, metastasis and stemness, which can ultimately lead to cancer relapse after primary treatment [1, 2, 7, 8, 9, 10, 11, 12, 13, 14, 15]. Cells may achieve polyploidy through genomic duplication or cell fusion events, but also through failures of chromosomal segregation during mitosis or in cytokinesis [16]. This leads to a range of genomic copy number variations and aneuploidy in cancer cells [16]. These circumstances result in many tumours (74% of polyploid tumours) harbouring a near‐triploid genome that can affect both disease progression and treatment outcome.

It has been previously shown that polyploidy has a role in cancer resistance to different chemotherapies and especially to genotoxic drugs. This likely arises from multiple copies of a given DNA segment serving as a template for homologous recombination‐driven DNA damage repair [8], and the presence of unmutated redundant copies of crucial genes remaining functional [17]. Cancer cell lines (CCLs), originating from specific cancer types, can be used to model the effect of a given anticancer drug in a specific context; however, due to cancer heterogeneity between patients and even within a tumour, it is difficult to address the effect of ploidy on treatment efficacy. These cell lines harbour different genetic and epigenetic mutations, and different aneuploidies that can affect experimental results and their interpretations [8]. Efforts to assess the impact of whole‐genome doubling (WGD) on the response to treatment have been made by comparing near‐diploid CCLs to their WGD‐induced, near‐tetraploid, counterparts [17]. However, this was done in only a few cancer types, and the application of these findings to different cancers should be explored further [17].

To examine the impact of ploidy on the response to anticancer drugs, we chose to investigate it in hESCs, which share many of the characteristics of malignant cells [18, 19]. Importantly, hESCs and CCLs also respond similarly to many chemotherapies [20]. In this study, we utilised isogenic haploid, diploid and triploid cell lines to explore the impact of ploidy, and especially triploidy, on the response to an array of anticancer treatments. In addition, as *TP53* is the most frequently mutated gene in human cancer [21, 22], and its well‐established involvement in the regulation of polyploid cell formation, proliferation and death [9, 17, 23, 24], we also mutated *TP53* in each of our cell lines and assessed the impact of ploidy in these hESCs. This study demonstrates the impact of ploidy on chemotherapy response in normal and *TP53*‐mutated cells.

## 2 Methods

### 2.1 Cell Culture

hESCs were handled under the Israeli guidelines regarding hESC research, and FAIR and CARE principles were followed. All cell lines were verified as haploid, diploid and triploid by G‐banding karyotype [6] and/or flow cytometry sorting based on DNA content, close to the experimental procedures. All cells were cryopreserved and thawed as previously described [25]. Attempts to produce isogenic normal tetraploid hESC lines were unsuccessful as the cells tend to be aneuploid. hESCs were cultured on Matrigel‐coated plates (Corning), in mTeSR1 feeder‐free maintenance medium for pluripotent cells (STEMCELL Technologies, Cat #85850), supplemented with 50 units/mL penicillin, 50 μg/mL streptomycin. Cells were grown in a humidified incubator at 37°C and 5% CO_2_. Cells were passaged by trypsinization using Trypsin solution A without EDTA (Biological Industries, Cat# 03‐052‐1A), supplemented with ROCK inhibitor Y‐27632 (Gentaur Cat# A3008) 24 h after splitting. Cells were mycoplasma free. The cell lines used during this study are listed in Table S1.

### 2.2 Cell Viability Assay Upon Anticancer Drugs Treatments

hESCs were grown on 96‐well plates in triplicates, with 12,000 cells per well. For each drug, 10 drug concentrations were tested and cell viability was evaluated using three independent survival experiments, for each ploidy level. The drug in each concentration was added on Day ‘0’ and the medium was replaced every 24 h. After 48 h of treatment, cell viability was assessed by the CellTiter‐Glo 2.0 Cell Viability Assay according to the manufacturer's instructions (Promega, Cat# G7570) and was monitored following 2, 3 and 5 days. Luminescence reads for each chemotherapy were normalised to control conditions (mTeSR without chemotherapies of every sample in each time point), and the replicate experiments were averaged. The mean intensity levels of each sample and different concentrations of chemotherapies were used to produce the survival curves and analyse the IC_50_ and AUC values of each cell line. For every survival curve, a second‐degree polynomial regression model was generated, from which both IC_50_ values and the AUC were calculated. Mean IC_50_ values of representative time‐points and mean AUC values of all time‐points, representative of cumulative survival, were compared between haploid, diploid and triploid WT hESCs, as well as *TP53*‐KO hESCs, using a one‐sided student's *t*‐test and *p* values were corrected according to the FDR method for multiple comparisons.

### 2.3 Apoptosis Analysis

hESCs were incubated in 37°C with mTeSR1 growth medium (STEMCELL Technologies, Cat #85850), either supplemented with bleomycin (40 nM, Selleck Chemicals, S1214), paclitaxel (3.3 nM, Cayman Chemical, 10461), cisplatin (100 nM, Cayman Chemical, 13119) or 5‐FU (5 μM, Cayman Chemical, 14416), or left untreated for 48 h. As described previously [6], these cell lines express either EGFP, DsRed or both, which their excitation‐emission wavelengths overlap those of propidium iodide (PI) and the Annexin‐FITC antibody, respectively, that are commonly used. So, to overcome this we used an Annexin‐V antibody conjugated to iFluor 555 (Annexin V‐iFluor 555, Abcam, ab219905) instead of the Annexin‐FITC antibody and performed the experiment using the spectral Cytek Aurora analyser. This device excites the cells with all of its lasers and measures the resulting emission of the cells to each of the lasers, thus enabling the identification of each fluorophore by its unique excitation‐emission signature. The cells were washed with phosphate buffered saline (PBS) with no calcium and magnesium, gently dissociated using Trypsin solution A without EDTA (Biological Industries), and then subjected to the MEBCYTO Apoptosis kit (CODE No. 4700) protocol, adjusted and calibrated for hESCs and for the usage of Annexin V‐iFluor 555 Apoptosis Staining/Detection Reagent instead of the Annexin‐FITC antibody (1.25 μL of PI and 1 μL of Annexin V‐iFluor 555 in 85 μL of the kit's Binding Buffer). The cells were then analysed using the spectral 4‐laser Cytek Aurora analyser to quantify the percentage of dead cells (positive for PI) and apoptotic cells (positive for Annexin V‐iFluor 555). Calibration was done by flow cytometry experiments of untreated diploid and triploid hESCs (negative controls) and diploid and triploid hESCs treated for 48 h with 1% DMSO (positive controls). Multiple titration experiments were done prior to the apoptosis experiment to calibrate the PI and Annexin V‐iFluor 555 antibody concentrations. Autofluorescence of the cells and the emission overlaps were compensated by setting a unique scaling factor for pES10 hESCs and by performing unmixing using each sample's control (group‐specific unstained control), respectively.

In the experiments, negative and positive thresholds were set using hESCs treated with the different chemotherapies that were not stained with PI nor with Annexin V‐iFluor 555, setting uniform thresholds for both PI and Annexin V‐iFluor 555 for all samples. We then used the FlowJo v10.8 Software (BD Life Sciences) to obtain the distribution of cells in each sample to either one of the quadrants (double negative, positive for Annexin V‐iFluor 555, positive for PI or double positive). The evaluation of apoptosis was done by comparing the apoptotic cell percentage (Annexin V‐iFluor 555 positive and double positive cells) between diploid and triploid hESCs in each treatment using a one‐sided student's *t*‐test, and *p* values were corrected according to the FDR method.

### 2.4 DAPI Staining and Nuclear Morphology Assessment

Diploid and triploid hESCs were grown on cover slips for 72 h with or without the addition of bleomycin (40 nM), paclitaxel (3.3 nM), cisplatin (100 nM) or 5‐FU (5 μM). Cells were then washed with PBS, fixed with 4% formaldehyde for 10 min at room temperature (RT), and then permeabilized using 0.5% Triton X‐100 in PBS for 20 min at RT. Next, the cells were washed with PBS and DNA was stained using 4′,6‐diamidino‐2‐phenylindole (DAPI, 1 μg/mL) for 10 min in the dark. Cover slips were then flipped over slides and were mounted and sealed using Immu‐Mount (Epredia). Images were taken using a fluorescent confocal microscope, 9 fields for each condition and sample (2 diploid and 3 triploid cell lines). Nuclei were identified using ImageJ (for detailed pipeline see the attached Macro script at Github), surface areas were measured using the ‘Analyze particle’ tool and nuclear volumes were calculated as 4/3*πr* ^3^. Analysis included more than 1000 nuclei per sample for each treatment. We tested the differences in nuclear size using a two‐sided student's *t*‐test, followed by *p* values correction by the FDR method.

### 2.5 53BP1 Immunofluorescent Staining and Foci Number Analysis

Diploid and triploid hESCs were grown on cover slips to a confluence of 70%–80% and then were treated for 1 h with bleomycin (2.5 μM) to induce DNA damage and then were grown in normal conditions to allow recovery for 24 h post‐treatment. Next, the cells were washed with PBS, fixed with 4% formaldehyde for 10 min at RT, and permeabilized with 0.5% Triton X‐100 in PBS for 20 min at RT. Cells were then stained with a primary antibody against p53‐binding protein 1—53BP1 (rabbit anti‐53BP1, 1:250), followed by staining with a secondary antibody to allow detection (donkey anti‐rabbit, Abcam, Alexa Fluor 647 ab150063, 1:500) and DAPI (1 μg/mL). Cover slips were then flipped onto slides, mounted and sealed using Immu‐Mount (Epredia). Images were taken using a fluorescent confocal microscope, 10 fields for each ploidy state. Nuclei and foci were identified using ImageJ (for detailed pipeline see the attached Macro script at Github), analysing 733 and 261 nuclei of treated diploid and triploid cells, respectively. Each measurement (nucleus) was assigned to a specific DNA damage foci number group to generate pie charts, and the relative mean number of DNA damage foci per cell was compared between diploid and triploid cells by a two‐sided student's *t*‐test.

### 2.6 DNA and RNA Purification

DNA extraction was done using the gSYNC DNA Extraction Kit (Geneaid). Purification of total RNA was done using the Qiagen Inc. RNEASY MINI KIT or using the MACHEREY‐NAGEL GmbH & Co KG NucleoSpin RNA Plus kit.

### 2.7 RNA‐seq

RNA samples were obtained from three samples of diploid pES10 cells (from two different cell lines) and three samples of triploid pES10 cells (from three different cell lines), treated with 20 nM of bleomycin, 3.3 nM of paclitaxel, 100 nM of cisplatin or 2.5 μM of 5‐FU for 48 h (total of 24 hESCs), were sent to The Center for Genomic Technologies of The Hebrew University of Jerusalem. We assessed the RNA quality by Bioanalyzer analysis (Nano‐kit). RNA samples (1–2 μg, RNA integrity number (RIN) > 9) were enriched for mRNAs by pulldown of poly(A)^+^ RNA and libraries were prepared using Illumina TruSeq RNA Library Prep Kit v2 (Cat# RS‐122‐2001). Sequencing was performed using NextSeq500 (Illumina) to generate 75 bp single‐end reads. For gene expression analysis, reads were mapped to the GRCh38 reference genome using STAR [26].

### 2.8 Data Acquisition and Normalization

Analysis of RNA‐seq data required additional read counts tables of untreated diploid and triploid hESCs. Hence, we used RNA‐seq data of four diploid and seven triploid pES10 hESCs from our previous study [6], available at the Array Express Archive of Functional Genomics Data (https://www.ebi.ac.uk/arrayexpress/) [27] under the accession number E‐MTAB‐11430. The rest of the RNA‐seq data was acquired during this study. All expression data were normalised together using the edgeR TMM normalisation procedure, as described below. IC_50_ data of CCLs were acquired from the Genomics of Drug Sensitivity in Cancer (GDSC) Project [28]. We also obtained the ploidy estimation of each cell line from the Catalogue Of Somatic Mutations In Cancer (COSMIC) [29]. In addition, data regarding the mutation in each cell line were obtained from the DepMap [30] portal (available at https://depmap.org/portal).

### 2.9 Differential Expression Analysis

After obtaining read counts tables of each sample, we performed a differential expression (DE) analysis using the edgeR package. The unified read counts tables were transformed into a digital gene expression (DGE) object by the DGEList function and then normalised by the calcNormFactors function. This procedure, known as a TMM normalisation, was used to acquire normalised log_2_(CPM) values for each gene in each sample. These values were used to compare gene expression between triploid and diploid hESCs (diploidy was considered the natural state), either treated or untreated with one of the chemotherapies. This was performed by the glmFit function, provided with a matrix of likelihoods produced by the estimateDisp function and a numeric design matrix (the factor we test for DE, i.e., ploidy) provided by the model.matrix function. The glmFit function fits the model to the dispersion, produces an object of class DGEGLM, which is passed to glmLRT function and later on to the topTags function to create a data‐frame of DE genes.

### 2.10 Gene Set Enrichment Analysis

After DE analysis was done, we assigned a rank to each gene according to its fold‐change (FC) in triploid cells compared to diploid cells, for each of the treatments. Ranks were calculated by multiplying each gene's sign(log_2_(FC)) by its −log(*p* value). These ranks were passed to the fgseaMultilevel function of the fgsea package in R, which assessed the enrichment of gene sets based on collections from the GSEA‐MSigDB [31]. The enrichment or depletion of each term's visualisation was done by multiplying the −log_2_(FDR) by the sign (+/−) of the normalised enrichment score (NES). The FDR threshold of 0.05 was marked to appreciate each term's significance.

### 2.11 Principal Component Analysis

Principal component analysis (PCA) was generated based on the normalised log_2_(CPM) values by using the prcomp function in R.

### 2.12 Germ Layer Specific Gene Expression Analysis

Genes specifically expressed in each of the three embryonic germ layers were previously defined [32]. To test the differentiation phenotype observed in our cells, we focused only on genes that are highly specific for their assigned germ layer (log_2_(fold change) > 5). The expression of these genes was examined in both WT and *TP53*‐KO hESCs and the expression levels (CPM) in triploid cells were normalised to the expression levels of each gene in the diploid hESCs for each one of the chemotherapies used in this study. The cell lines used for the classification of the germ layer specific genes can be found in the Supporting Information of the article [32], and the list of genes and their germ layer specification are in Table S3.

### 2.13 *TP53* Knock‐Out and *TP53* ‐KO Cells Selection

Haploid, diploid and triploid hESCs were transiently transfected using the X‐tremeGENE 9 DNA Transfection Reagent with pSpCas9(BB)‐2A‐GFP (PX458) vector (a gift from Feng Zhang, Addgene cat. #48138) with a gRNA directed against *TP53* (5′‐GACGGAAACCGTAGCTGCC‐3′) according to manufacturer instructions, as described in a previous study from our lab [33]. We validated the transfection success 48 h post transfection by testing for GFP‐positive cells in our mass cultures (only possible with the DsRed‐only‐positive cells as the rest express EGFP already). Cells were then treated for ≥ 10 days with Nutlin‐3a to facilitate enrichment of *TP53*‐null cells.

### 2.14 *TP53* ‐KO Validation in hESC Lines

To verify mutations in the *TP53* gene, we extracted DNA from the WT and mutant cells and performed Sanger sequencing of the *TP53* editing site using forward primer: 5′‐GAGGGTGTGATGGGATGGAT‐3′ and reverse primer: 5′‐AGAACGTTGTTTTCAGGAAGTCT‐3′. To infer on the *TP53* editing efficacy, fastq file of each cell line was uploaded to the TIDE (Tracking of Indels by DEcomposition) website [34].

### 2.15 DNA Damage Response Analysis in *TP53* ‐KO hESCs

Analysis of DNA damage response‐related terms in GSEA (the LeadingEdge) enabled us to focus on genes directly linked to the ‘DNA repair’ pathway. We compared the normalised expression levels of these genes in *TP53* ‐KO triploid hESCs to diploid *TP53*‐KO cells, and utilising the pheatmap function showed a heatmap of each gene's expression ratio.

### 2.16 CCLs Sensitivity Assessment to Chemotherapies

IC_50_ data of CCLs were acquired from the GDSC database [28] and to each cell line we assigned its ploidy estimation from COSMIC [29]. We collected hundreds of CCLs' IC_50_ data treated with the four chemotherapies we used during this study, as well as the status of *TP53* in these CCLs. CCLs with an average ploidy of 2.5 or lower were considered near‐diploids, and CCLs with an average ploidy higher than 2.5 and lower than 3.5 were regarded as near‐triploids. CCLs which completely lost their WT *TP53* alleles were considered *TP53*‐deficient (*TP53* full LoF). The comparisons between the different ploidy groups as well as their *TP53* status were performed by a one‐sided *t*‐test on the IC_50_ values of each cell line to a given chemotherapy. The ploidy level and *TP53* status of each CCL are listed in Table S2.

## 3 Results

### 3.1 Assessment of Cell Survival Response to Chemotherapies

We recently established several isogenic haploid, diploid and triploid hESC lines [5, 6, 35], and here, we utilise multiple and distinct lines of these cells to test the effect of triploidy on the response to chemotherapies with different mechanisms of action that are prevalent in clinical practice: bleomycin, paclitaxel, cisplatin and 5‐fluorouracil (5‐FU). Bleomycin causes oxidative DNA damage by binding to metal ions, causing both single‐ and double‐strand breaks [36]. Paclitaxel interferes with the normal function of microtubules (microtubule stabiliser), which leads to G2/M cell cycle arrest. Interestingly, paclitaxel can also induce DNA damage, but this effect is less characterised [37, 38]. Cisplatin crosslinks DNA, interfering with DNA replication, transcription, mitotic cell division and by inducing DNA repair mechanisms, leading to apoptosis [39, 40]. 5‐FU inhibits thymidylate synthase (TS) and also incorporates into RNA and DNA and thus disrupts DNA and RNA function [41].

**FIGURE 1:**
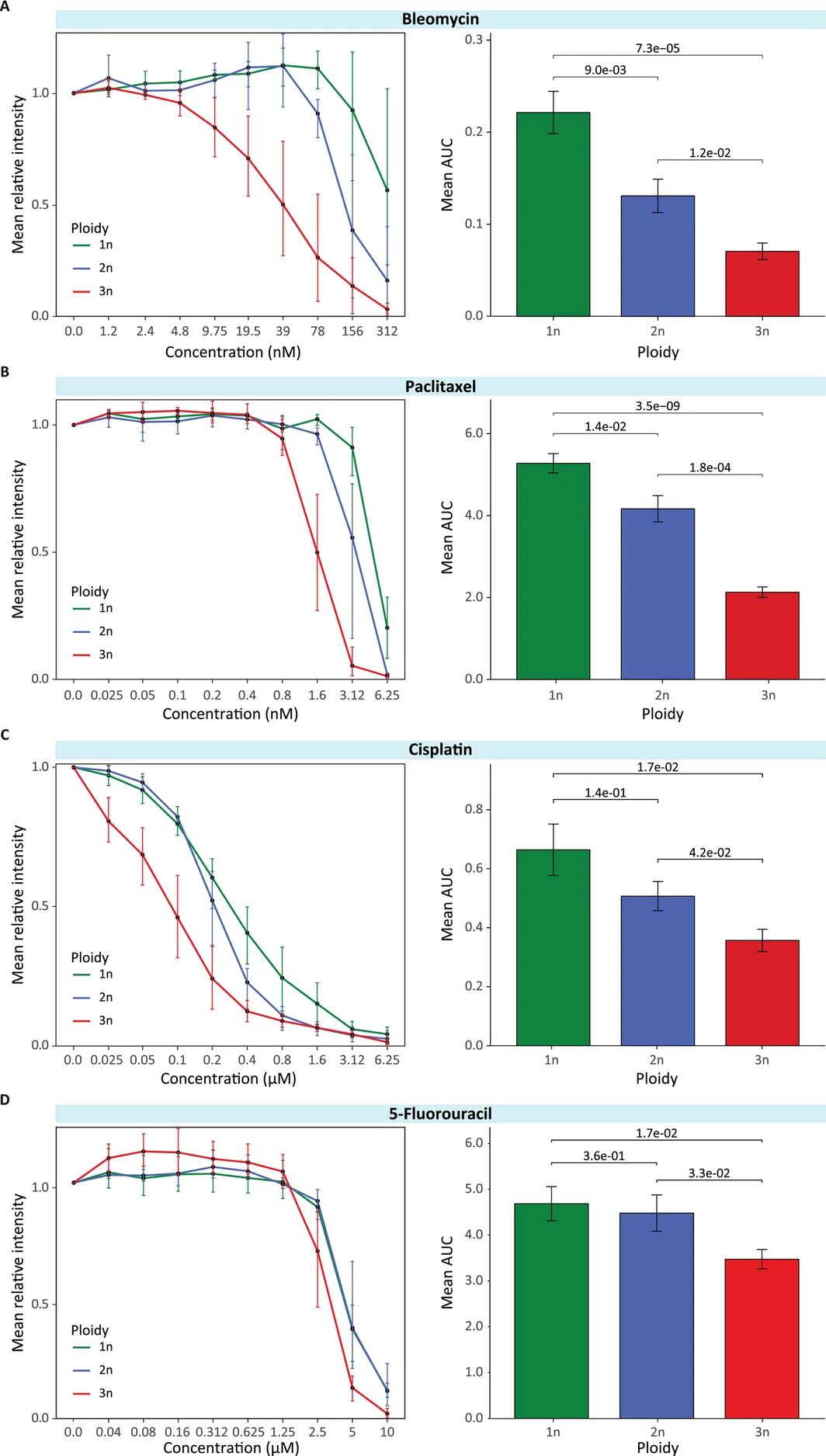
Survival assay and sensitivity assessment by AUC of chemotherapies‐treated hESCs with different ploidies. (A–D) The left panel in each subset is the CellTiter‐Glo mean relative intensity levels, showing the survival of haploid, diploid and triploid hESCs. The X‐axis is on a logarithmic scale. The right panel are mean AUC values, calculated from AUC values of day 2, 3 and 5 after exposure to different concentrations of bleomycin (A), paclitaxel (B), cisplatin (C) and 5‐FU (D). The comparison was done by performing a student's *t*‐test, with FDR‐corrected *p* values. Error bars represent the standard error of the mean (SEM). *n* = 4, 3 and 5 for the haploid, diploid and triploid cells, respectively.

First, we conducted survival assays of haploid, diploid and triploid hESCs by exposing them for 48 h to different concentrations of each of the anticancer drugs mentioned above, evaluating cell survival after 48, 72 and 120 h. Survival for each drug was quantified using the CellTiter‐Glo 2.0 Cell Viability Assay and followed by calculating area‐under‐the‐curve (AUC) for survival data and the inhibitory concentration where 50% of the cells died (IC_50_). For all four chemotherapies, the mean AUC values of the triploid hESCs were significantly lower than those of the haploid and diploid cells, and upon bleomycin and paclitaxel treatments, the cells exhibited decreased cell survival as ploidy increased from haploid to diploid and triploid (Figure 1). The IC_50_ values of triploid hESCs were also significantly lower than those of haploid and diploid cells for three out of the four chemotherapies (Figure S1A–D). 5‐FU induced a similar trend but showed a significant difference only between haploid and triploid hESCs' IC_50_ values. These results demonstrate that ploidy significantly affects the cellular response to anticancer drugs and emphasize the effect of triploidy. For the various chemotherapies, the survival curves of triploid cells differ from those of haploid and diploid hESCs in nearly all of the timepoints we tested (Figure 1).

### 3.2 Analysis of Apoptosis in Diploid and Triploid Cells

**FIGURE 2:**
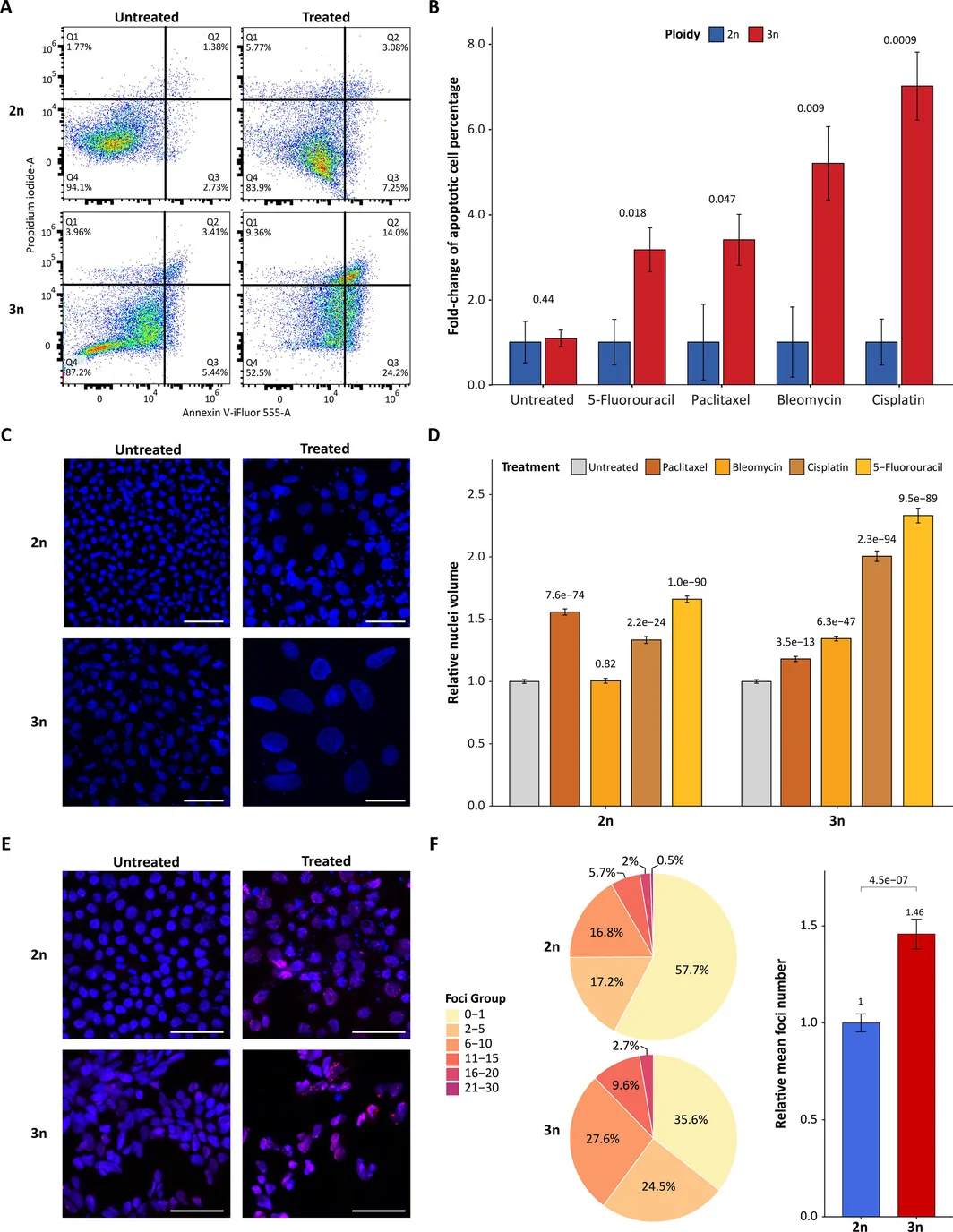
Apoptosis and nuclear morphology of hESCs treated with chemotherapies. (A) Representative flow‐cytometry plots of diploid (top) and triploid (bottom) hESCs untreated (left) or treated with cisplatin (100 nM, right) after apoptosis assay was performed. The analysis was done using the spectral Cytek Aurora flow cytometer. (B) Fold‐change of the percentage of apoptotic cells for each treatment, relative to the percentage of apoptotic cells in diploid hESCs cultures. The comparison was done by performing a one‐sided student's *t*‐test. Error bars represent SEM. *n* = 3 for both diploid and triploid hESCs. (C) Representative images of diploid (top) and triploid (bottom) hESCs either untreated (left) or treated with 5‐FU (5 μM, right) for 72 h and then stained with DAPI. Scale bar = 50 μm. (D) Relative nuclear volume of diploid and triploid hESCs either untreated or treated with bleomycin (40 nM), paclitaxel, (3.3 nM), cisplatin (100 nM) and 5‐FU (5 μM) for 72 h. Nuclei areas were measured, nuclei volumes were calculated and then divided by the mean nuclear volume of untreated cells, for each ploidy state. *n* > 1000 for each treatment and each ploidy state. The comparison was done by a two‐sided student's *t*‐test. Error bars represent SEM. (E) Representative images of diploid (top) and triploid (bottom) hESCs either untreated (left) or treated with bleomycin (2.5 μM, right) for 1 h and then stained with DAPI and antibody against 53BP1 24 h post‐treatment. Scale bar = 50 μm. (F) Pie charts representing the percentage of cells with different ranges of foci number in diploid (top) and triploid (bottom) hESCs (left panel) and the mean number of foci per cell in diploid and triploid hESCs (right panel), treated with bleomycin (2.5 μM) for 1 h, stained with DAPI and antibody against 53BP1 after 24 h recovery.

To validate the differences in the survival assay results between the diploid and triploid hESCs, and to quantify the cell death induced by the different treatments, we performed apoptosis assays following 48 h of exposure to bleomycin, paclitaxel, cisplatin and 5‐FU. Subsequent analysis revealed a significantly increased apoptosis rate in triploid compared to diploid cells in all treatments, with cisplatin having the strongest effect of approximately seven‐fold higher apoptotic cells' percentage (Figures 2A,B and S1E). These results reinforce the notion that differential ploidy levels confer altered sensitivity of cells to chemotherapy.

### 3.3 Chemotherapies Affect the Nuclear Size of Triploid Cells

As some chemotherapies, including the ones in this study, were shown to interfere with the progression of the cell cycle and ultimately increase the ploidy of cells post‐treatment [39, 42, 43, 44, 45, 46, 47], we analysed the nucleus size of our cells before and after treatment. Diploid and triploid hESCs were exposed to all four chemotherapies for 72 h, stained with DAPI and nuclei sizes were evaluated by fluorescent confocal microscopy. Nuclear area was measured directly; however, the nuclear volume was assessed as if the nuclei are spherical, an assumption that might influence the results. Nevertheless, the difference in nuclear size we observed was prominent, with the most pronounced effect of chemotherapy in triploid versus diploid cells observed in cells treated with 5‐FU: diploid nuclei size was 1.65‐fold larger in the drug‐exposed cells, while treated triploid nuclei were 2.33‐fold larger than their control counterparts (Figure 2C,D). Bleomycin induced a small change in the nuclear size of diploid hESCs, while triploid cells exhibited a 1.34‐fold increase in their mean nuclear size (Figures 2D and S1F, top). Cells treated with cisplatin also displayed a significant increase in their nuclear size, with treated diploid nuclei that were 1.33‐fold larger and treated triploid nuclei that were twice the size of their control counterparts (Figures 2D and S1F, middle). Conversely, diploid cells treated with paclitaxel exhibited a 55.5% increase in their mean nucleus size, while triploid cells that were exposed to paclitaxel had only an 18% increase in their mean nucleus size compared to untreated cells (Figures 2D and Figure S1F, bottom). These findings suggest that our hESCs experience cell cycle arrest and increased ploidy following treatment with one or more of the chemotherapies we used, recapitulating the response of somatic and malignant cells, and point to the impact of ploidy on cell death and nuclear morphology.

### 3.4 Triploid Cells Experience Higher Levels of DNA Damage in Response to Chemotherapy

As triploid cells harbour an extra chromosomal set, we hypothesised that in response to genotoxic insult, they will acquire ~1.5‐fold more DNA damage compared to diploid cells. Therefore, to test this notion, we treated diploid and triploid hESCs for 1 h with bleomycin to induce DNA damage, and then we performed immunofluorescent staining with an antibody against 53BP1, which is a marker of DNA damage foci [48, 49] (Figure 2E). We then quantified the number of DNA damage foci per cell in both diploid and triploid hESCs and noticed that in diploid cells, most of the cells (57.7%) had between 0 and 1 focus, and the rest of the cells had 2 or more foci in them (Figure 2F, top left). However, the majority of triploid cells (~65%) contained 2 or more foci, while only ~35% of them had between 0 and 1 focus of DNA damage (Figure 2F, bottom left). We also compared the mean foci number per cell between diploid and triploid hESCs post‐treatment and found that the triploid cells had a 1.46‐fold higher mean foci number than their diploid counterparts, supporting our hypothesis that triploid cells have a higher level of DNA damage when compared to diploid cells (Figure 2F, right).

### 3.5 The Involvement of p53 in Cell Fate Determination Upon Chemotherapy Treatment

**FIGURE 3:**
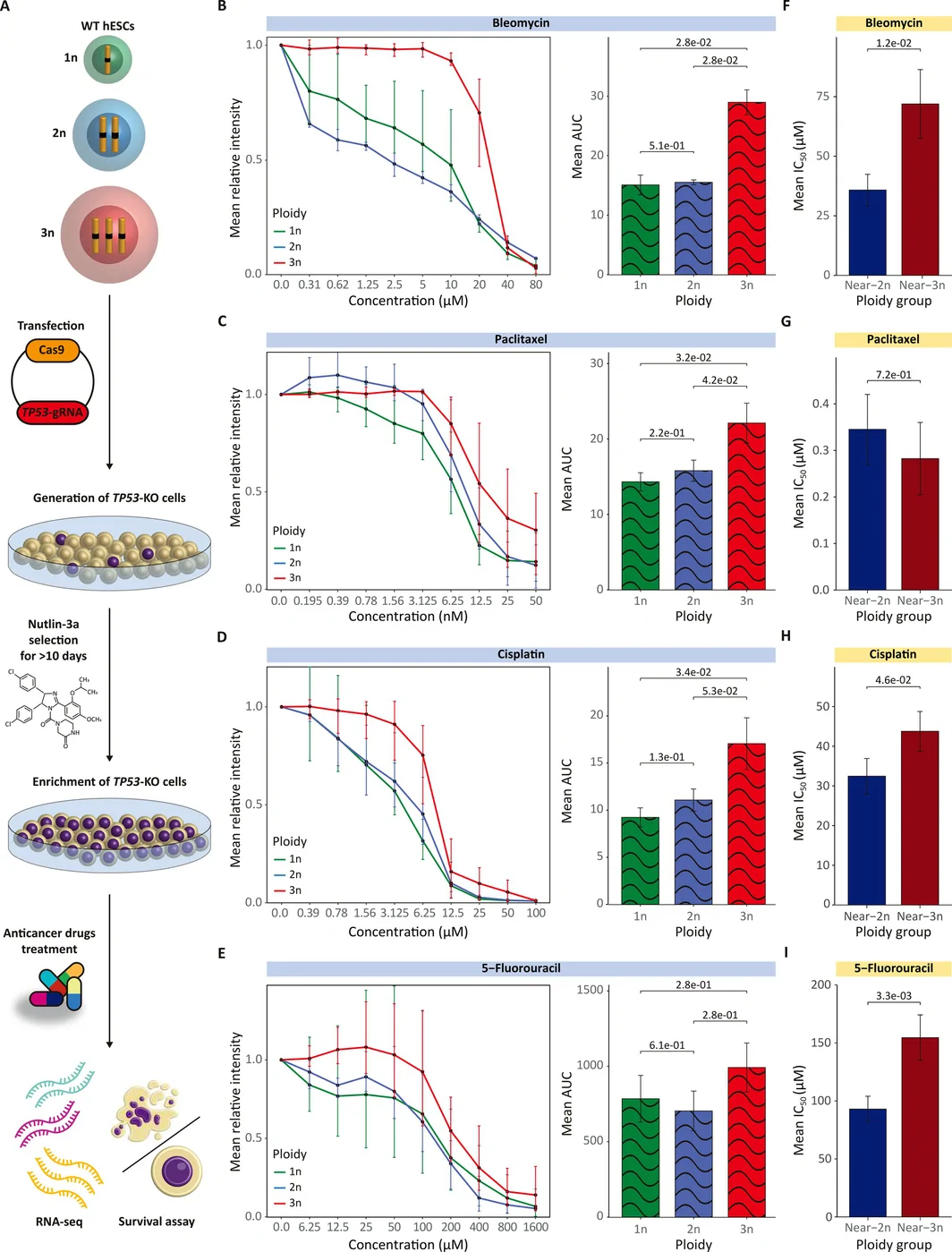
Survival assay and sensitivity assessment of treated *TP53*‐KO hESCs and CCLs with different ploidies. (A) An illustration of the process of generating *TP53*‐KO hESCs and their enrichment in culture by nutlin‐3a treatment. Cells with purple nuclei represent *TP53*‐KO cells. (B–E) A comparison between the sensitivities of *TP53*‐null hESCs with different levels of ploidy. Left panel are mean AUC values, calculated from AUC values of day 2, 3 and 5 after exposure of *TP53*‐null hESCs to different concentrations of bleomycin (A), paclitaxel (B), cisplatin (C) and 5‐FU (D). The comparison was done by performing a one‐sided student's *t*‐test, with FDR‐corrected *p* values. The right panel in each subset is the CellTiter‐Glo mean relative intensity levels, showing the survival of haploid, diploid and triploid *TP53*‐null hESCs from the most representative timepoint. *n* = 3 for both diploid and triploid hESCs. The X‐axis is on a logarithmic scale. (F–I) Sensitivity assessment of CCLs to each chemotherapy according to their IC_50_ values obtained from the GDSC. The comparison was done by performing a one‐sided student's *t*‐test. Error bars represent SEM. For bleomycin (F), paclitaxel (G), cisplatin (H) and 5‐FU (I) *n* = 244, 151, 237 and 251 for near‐diploid CCLs and *n* = 259, 128, 238 and 269 for near‐triploid CCLs, respectively.

As mentioned above, the death of the cells treated with anticancer drugs was due to a higher apoptosis rate, especially in the triploid cells. p53 is the main regulator of cell apoptosis [50, 51]. Therefore, to explore the involvement of *TP53* in the ploidy‐dependent response to the anticancer drugs, we generated haploid, diploid and triploid hESC lines with *TP53* loss‐of‐function mutations by transfecting them with a vector containing the Cas9 coding sequence and a *TP53* guide RNA (gRNA) sequence (Figure 3A). Two days post transfection, the cells were treated with nutlin‐3a, a highly specific small molecule that binds MDM2 in the p53‐binding pocket, preventing p53 degradation by the proteosome. Thus, nutlin‐3a causes the accumulation of p53 in the cells and ultimately leads to apoptosis [52]. The cells were exposed to this treatment for more than 10 days, enabling us to select for *TP53*‐null hESCs (Figure 3A), which was validated using Sanger sequencing followed by TIDE decomposition assessments of CRISPR‐Cas9 efficacy in each cell line (Figure S2A).

Once obtaining haploid, diploid and triploid *TP53*‐knock‐out (KO) cultures, we exposed them to different concentrations of the same chemotherapies and assessed their survival. As expected, *TP53*‐null cells demonstrated considerable resistance to all chemotherapies. Therefore, we had to increase the concentrations to reach their IC_50_ values (Figures 3B–E and S2B–E). Haploid and diploid *TP53*‐null hESCs demonstrate similar sensitivity to all four chemotherapies, but interestingly, triploid *TP53*‐null hESCs display a higher resistance to all chemotherapies in comparison to their haploid and diploid counterparts (Figure 3B–E). The effect was significantly higher in three out of the four chemotherapies. This trend was also observed when we compared the IC_50_ values between haploid, diploid and triploid *TP53*‐KO hESCs and was significantly demonstrated in the bleomycin‐treated cells (Figure S2B–E). Furthermore, the inversion of the sensitivity phenotype between WT and *TP53*‐null cells indicates that p53 is involved in the ploidy‐dependent response to anticancer drugs.

### 3.6 Cancer Cell Lines Response to Anticancer Drugs

As tumours and their derived cancer cell lines (CCLs) are extremely heterogenous, there is no available data regarding the sensitivity of isogenic near‐diploid and near‐triploid cancer cells. Therefore, to assess our capability to model triploidy in cancer, we obtained IC_50_ data from the Genomics of Drug Sensitivity in Cancer (GDSC) Project [28] of all the different CCLs exposed to the four chemotherapies we used in our study. We also assigned each cell line with its ploidy estimate from the Catalogue Of Somatic Mutations In Cancer (COSMIC) [29] as well as their *TP53* status (Table S2), sorting the CCLs into near‐diploids and near‐triploids and their *TP53* function, then comparing their IC_50_ values for each of the anticancer drugs. We show that malignant cells responded similarly to our *TP53*‐KO hESCs upon bleomycin, cisplatin and 5‐FU treatments (Figure 3F,H,I). On the other hand, near‐diploid and near‐triploid CCLs treated with paclitaxel had no significant difference between their IC_50_ values (Figure 3G). Although it possesses some DNA‐damage capability [53], paclitaxel is the only anticancer drug we used whose primary mechanism is microtubule disruption [38], which could also explain why it is the only one that does not induce a similar response in all CCLs. While cancer cells can acquire alterations in the expression or function of DNA repair‐related genes, enabling them to endure the accumulating DNA damage induced by all four chemotherapies, they can also obtain changes in the tubulin genes themselves and their related proteins (mostly to β‐Tubulin) [54], which might be unique to paclitaxel and its derivatives and are not affected by the ploidy of the cells. Furthermore, subdividing CCLs into *TP53*‐proficient (harbouring at least one intact copy of *TP53*) and *TP53*‐deficient (full loss of function) lines, followed by a comparison between near‐diploids and near‐triploids CCLs in each group revealed a trend where *TP53*‐deficient near‐triploids CCLs are ~1.5–2 times more resistant than *TP53*‐deficient near‐diploids CCLs (Figure S2F–I). Conversely, both near‐diploid and near‐triploid *TP53*‐proficient CCLs displayed similar sensitivity to all chemotherapies (Figure S2F–I), highlighting the impact of full *TP53* Loss of function (LoF) on the ploidy‐dependent sensitivity to different treatments. Overall, the phenotype we observed in *TP53*‐null hESCs corresponds to the resistance of near‐triploid cancer cells to various treatments, especially in the context of *TP53* LoF.

### 3.7 Gene Expression Analysis of Treated hESCs

**FIGURE 4:**
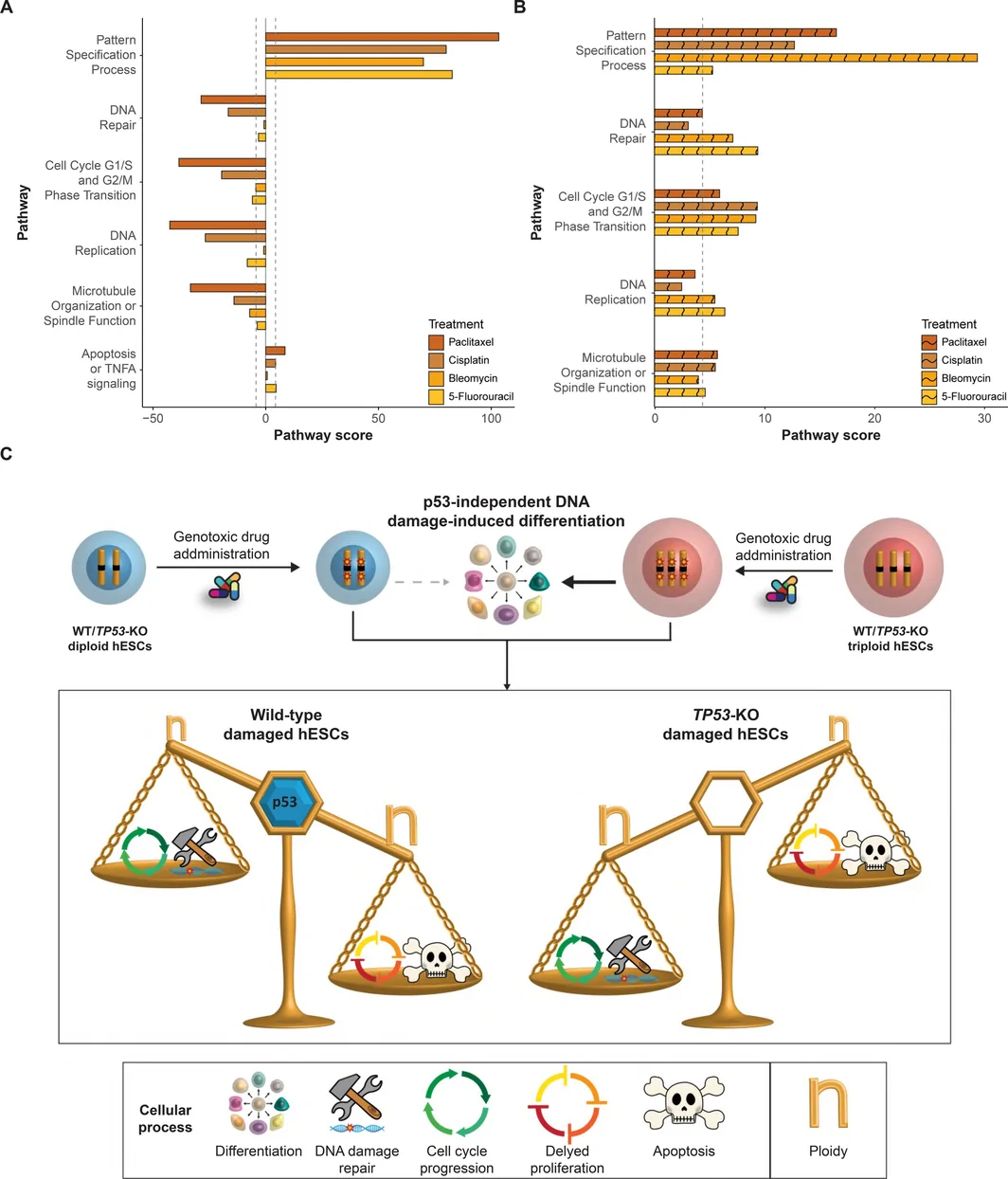
Transcriptome analysis of WT and *TP53*‐KO treated hESCs and a model of the ploidy‐dependent response to chemotherapy. (A, B) Gene set enrichment analysis showing terms associated with gene sets that were significantly changed in WT (A) and *TP53*‐KO (B) treated triploid hESCs compared to their diploid counterparts. The score was calculated by multiplying the −log_2_(FDR) by the sign of the normalised enrichment score (NES). The dashed line indicates the FDR threshold for significance. For both WT and *TP53*‐KO cells, *n* = 3 for each of the diploid and triploid hESCs treated with either one of the chemotherapies. (C) A schematic representation of the proposed model of the ploidy‐dependent response to anticancer drugs. Irrespective of p53 status, treated triploid hESCs undergo DNA damage‐induced differentiation much more than treated diploid cells (top). When p53 is functional DNA damage accumulates due to genotoxic drug administration, leading to higher absolute number of DNA damage foci (~1.5‐fold higher) in the triploid cells. hESCs with higher ploidy (indicated by the size of the “*n*” weight) are much more likely to reach the p53‐mediated threshold for DNA damage, which sets off a cell cycle arrest and apoptotic commitment, while cells with lower ploidy have a better chance of repairing the damage and continue to proliferate (bottom‐left). When p53 is absent hESCs with more genomic copies can endure the excess damage, probably due to higher number of unmutated alleles and reliance on homologous recombination‐driven DNA damage repair (bottom‐right).

To investigate the transcriptomic response of the cells to different treatments, we exposed WT diploid and triploid hESCs to each of the chemotherapies for 48 h, followed by RNA sequencing (RNA‐seq) analysis of the cells. PCA based on the gene expression signatures of both untreated and treated diploid and triploid cells revealed a clear separation between treated and untreated cells (PC1), and separation to some extent according to their ploidy state (PC2). Yet, untreated diploid and triploid cells were clustered very close to one another, while treated diploid cells clustered separately from the treated triploid cells, regardless of the chemotherapy they were exposed to (Figure S3A). The results suggest that triploid cells react differently than diploid cells to several anticancer drugs on a transcriptomic level. Hence, we performed gene set enrichment analysis (GSEA) of the WT diploid versus triploid cells upon exposure to chemotherapies. We revealed a unique transcriptional response of triploid cells common to all treatments, supporting the PCA results, but also identified other groups of pathways that were altered between diploid and triploid cells in only a subset of the chemotherapies (Figure 4A). Triploid hESCs were more likely to experience G1/S and G2/M checkpoint activation followed by cell cycle arrest, as well as mitotic failure (indicated by the downregulation of “Cell Cycle G1/S and G2/M Phase Transition” and “Microtubule Organization or Spindle Function” related pathways, respectively, Figure 4A). In addition to these terms, triploid hESCs displayed a robust differentiation phenotype in response to all chemotherapies, and remarkably, the chemotherapy‐induced differentiation (marked as “Pattern Specification Process”) was observed in both diploid and triploid cells, but was much more pronounced in triploid hESCs, both WT and *TP53*‐KO cells (Figure 4A,B). This effect is also reported to occur in different cancer types [42, 55, 56, 57, 58]. To support this observation, we tested the expression of genes that were identified as markers of the three embryonic germ layers [32] in our treated hESCs. Using genes that were highly specific for the differentiation to either one of the three germ layers (Table S3), we observed significantly elevated levels of these genes in treated triploid hESCs compared with their diploid counterparts in both WT and *TP53*‐KO cells (Figure S3B,C), indicating all chemotherapies induced the differentiation of these cells. WT triploid cells also exhibited relatively low expression levels of genes related to DNA replication and DNA damage response, as well as higher expression levels of genes related either to apoptosis or TNF‐α signalling pathways, in response to all chemotherapies except bleomycin (Figure 4A). Interestingly, performing GSEA on the transcriptomes of treated *TP53*‐KO cells revealed the opposite trend for all pathways, except the differentiation‐related pathways as mentioned above (Figure 4B). To validate the observed DNA damage response (DDR) upregulation in the *TP53* mutant triploid cells, we performed a STRING analysis of the top upregulated DDR‐related genes. This analysis revealed an enrichment of these genes in treated *TP53*‐KO triploid cells relative to diploid cells, that was statistically significant in three out of the four chemotherapies (Figure S4A) and an overall upregulation of DNA damage repair genes in mutant triploid cells (Figure S4B). The core DNA damage repair genes were highly connected, including canonical genes such as *RAD51B*, *ATM*, *ATR*, *BRCA2*, *MLH1*, *MLH3* and *MSH2* and *FANCC* and *FANCI*, and each gene was found to be enriched in triploid cells compared to diploid cells in at least one treatment, indicating the repair of DNA damage of different types (Figure S4C,D) [42, 55, 56, 57, 58]. These findings support our previous results and point to both p53‐dependent and p53‐independent response, impacted by the ploidy of the cells.

## 4 Discussion

In this study, we utilised isogenic hESC lines with different ploidy levels to explore the impact of ploidy, and more specifically triploidy, on the response of hESCs to various anticancer drugs. We assessed the viability of our cells in different concentrations of bleomycin, paclitaxel, cisplatin and 5‐FU, and to our surprise, we found that triploid hESCs were more sensitive to these chemotherapies than their haploid and diploid counterparts (Figures 1 and S1A–D). We also validated the apoptotic induction in these cells and observed a similar trend, as more triploid cells have undergone post‐treatment apoptosis compared to diploid cells, and we also noticed a significant increase in nucleus size after treatment, especially in triploid hESCs (Figures 2A–D and S1E,F). Overall, the anticancer drugs we used in this study induced a strong ploidy‐dependent phenotype, which for the most part is likely p53‐dependent. This notion is supported by the fact that a knockout (KO) of *TP53* reversed the ploidy‐dependent chemoresistance phenotype. As p53 was shown to regulate DNA damage and microtubule‐related cell death, such as apoptosis [24, 50, 59], it stands to reason that *TP53*‐KO rendered our cells much more resistant to chemotherapies. More importantly, it reversed the outcome and the ploidy‐dependent response to chemotherapies, leading to higher resistance of triploid cells compared to their diploid counterparts (Figures 3 and S2B–E). We also demonstrate that our hESCs recapitulate the effect of ploidy in CCLs (Figures 3 and S2F–I).

The transcriptomic analyses also support the apoptotic and morphological phenotypes we observed in our treated cells, as WT triploid hESCs exhibited both G1/S and G2/M transition failures, as well as lower levels of gene expression related to the progression and completion of mitosis when compared to diploid cells (Figure 4). This phenotype is likely caused by the p53‐mediated DNA damage surveillance system, sensing the higher absolute levels of DNA damage accumulating in triploid cells (Figure 2E,F), surpassing a certain threshold of DNA damage [60, 61], which then leads to cell cycle arrest and commitment to cell death for comparatively lower drug concentrations. Interestingly, triploid *TP53*‐KO cells are better able to endure the damage they experience, and they manage to proliferate relatively more post‐treatment, leading to better survival in comparison to diploid cells (Figure 4). We previously reported that our triploid hESCs differentiate poorly to the three germ layers and they remain pluripotent in vivo while their diploid counterparts easily form teratomas [6], a trait shared between them and polyploid cancer cells, which are often less differentiated than near‐diploid malignant cells [43, 44]. Interestingly, the cells undergo differentiation upon exposure to all four treatments, but this phenomenon was significantly elevated in both WT and *TP53*‐KO triploid hESCs. This points to the involvement of a DNA damage sensing process, perhaps independently of p53 status although other p53‐related genes may be involved, that triggers cell differentiation in response to the level of DNA damage, a phenomenon which has been reported previously [62, 63]. This notion implies that our triploid cells, both WT and *TP53*‐KO, are more susceptible to DNA damage‐induced differentiation than our diploid cells, as they acquire more DNA damage foci for a given concentration (Figures 2E,F and S3B,C).

We suggest the possibility that the DNA damage surveillance system is responsible for the differentiation of damaged cells, and it is triggered as a function of absolute levels of DNA damage and is therefore more likely to be activated as ploidy increases (e.g., in triploid cells harbouring 1.5‐fold more DNA and consequently 1.5‐fold more DNA damage than diploid cells, Figures 2E,F and 4C, top). Unlike the differentiation phenotype we observed, the cellular death and growth inhibition of DNA‐damaged cells is facilitated by p53, which in WT cells causes higher‐ploidy cells to undergo more apoptosis and cell cycle arrest (Figure 4C, bottom‐left). However, in *TP53*‐null cells, the absence of p53 causes the increased ploidy to tip the scales towards DNA damage repair and proliferation (Figure 4C, bottom‐right). In conclusion, in our research we could separate the state of pure polyploidy and polyploidy with *TP53* loss‐of‐function. While polyploidy itself promotes sensitivity to chemotherapy, polyploidy with elimination of p53 enables higher resistance.

Malignant cells harbour numerous point mutations, epigenetic alterations and chromosomal aberrations, leading to different effects on gene expression and cell cycle regulation, as well as possible changes in metabolism and DNA damage repair capabilities. However, our hESCs are karyotypically intact, have no mutations (other than the desired *TP53*‐KO) and behave similarly to other pluripotent cells. This study aims to underline the impact of triploidy on the sensitivity to anticancer drugs on a broader scale, but its implications to cancer should be further validated. Still, based on our experiments using hESCs and analyses of the sensitivity of CCLs, it is likely that the chemotherapies applied here will probably be less effective for treating cancer patients with near‐triploid malignant cells, especially if they carry a *TP53* LoF.

With this in mind, since polyploid cancer cells are usually more resistant to chemotherapy, and the fact that *TP53* or its downstream effectors are mutated in most cancers, it is crucial to model the effect of polyploidy in the context of *TP53* deficiency. In addition, the effect of certain chemotherapies on cancer cells may vary depending on their tissue of origin, their specific aneuploidies and genetic and epigenetic mutations they acquire. Therefore, given their intact karyotype, identical genomic background, imprinting signature and X chromosome activation status, our hESC lines can be used to study the effect of triploidy, with and without *TP53* mutation in the context of chemotherapy resistance, but translation into clinical implications requires further validation in authentic tumour samples.

Taken together, our study reveals the role of ploidy in determining cellular fate upon exposure to chemotherapy. This is probably executed via the DNA damage surveillance machinery, either in cooperation with p53, which decides whether DNA damage repair, proliferation or apoptosis should take place, or the probability of an exit from pluripotency, governed by the amount of damage the cells sense, independently of p53 function. This study opens the possibility to isolate and investigate the effects of ploidy and p53 status on the response to many more chemotherapies and alternative treatments in the future. Furthermore, it holds the potential to identify novel targets for polyploid giant cancer cells, a major obstacle which has yet to be overcome to prevent chemoresistance and tumour recurrence.

## Author Contributions

Conceptualization: G.H.‐A. and N.B. Methodology: G.H.‐A. and N.B. Investigation: G.H.‐A. Visualisation: G.H.‐A. Funding acquisition: N.B. Project administration: N.B. Supervision: N.B. Writing – original draft: G.H.‐A. and N.B. Writing – review and editing: G.H.‐A. and N.B.

## Funding

This work was supported by the Azrieli Foundation, the Rosetrees Trust, the Israel Science Foundation [2054/22], the ISF‐Israel Precision Medicine Partnership Program [3605/21], the United States‐Israel Binational Science Foundation [2021278], and the MRC grant UKRI1687, awarded to the Investigating Safety and Toxicity of Advanced Therapies (iSTAT) Prosperity Partnership.

## Ethics Statement

All experimental procedures were performed according to the ethical regulations of the Hebrew University of Jerusalem and comply with the ISSCR guidelines.

## Conflicts of Interest

The authors declare no conflicts of interest.

## Supporting information


**Figure S1:** Sensitivity assessment to anticancer drug exposure of hESCs with different ploidies.
**Figure S2:** Validation of CRISPR‐Cas9 efficacy targeting *TP53* and chemotherapy sensitivity assessment of *TP53*‐KO in hESCs and CCLs with different ploidy levels.
**Figure S3:** Separation of treated and untreated hESCs based on gene expression and differentiation assessment in treated hESCs.
**Figure S4:** DNA damage response of *TP53*‐KO hESCs upon exposure to anticancer drugs.

**Table S1:** Cell lines.

**Table S2:** Cancer cell lines data.

**Table S3:** Germ layers specific differentiation genes.

## Data Availability

The data that support the findings of this study are openly available in ArrayExpress Archive of Functional Genomics Data at https://www.ebi.ac.uk/arrayexpress/ [27]. RNA‐seq transcriptomic data of WT untreated hESCs are available under the accession number E‐MTAB‐11430 and RNA‐seq transcriptomic data of treated WT hESCs, as well as treated and untreated *TP53*‐KO hESCs data are available under the accession number E‐MTAB‐16158. Publicly available reference genomes GRCh38 and GRCm38 were used for analysis. All codes used for the analyses and figure generation are available at https://github.com/guy‐haim/Ploidy‐Dependent‐Cellular‐Fate‐of‐Human‐Embryonic‐Stem‐Cells‐in‐Response‐to‐Anticancer‐Drugs. The data that support the findings of this study are available from the corresponding author upon reasonable request.
