# Supplementary material for: Ploidy‐Dependent Response to Anticancer Drugs of Human Embryonic Stem Cells
Source: Cell Prolif. 2026 Sep 1:e70278. Online ahead of print. doi: 10.1111/cpr.70278 (PMC13533588; doi:10.1111/cpr.70278)
Supplement: Supplementary file 1 [file CPR-9999-e70278-s001.pdf]

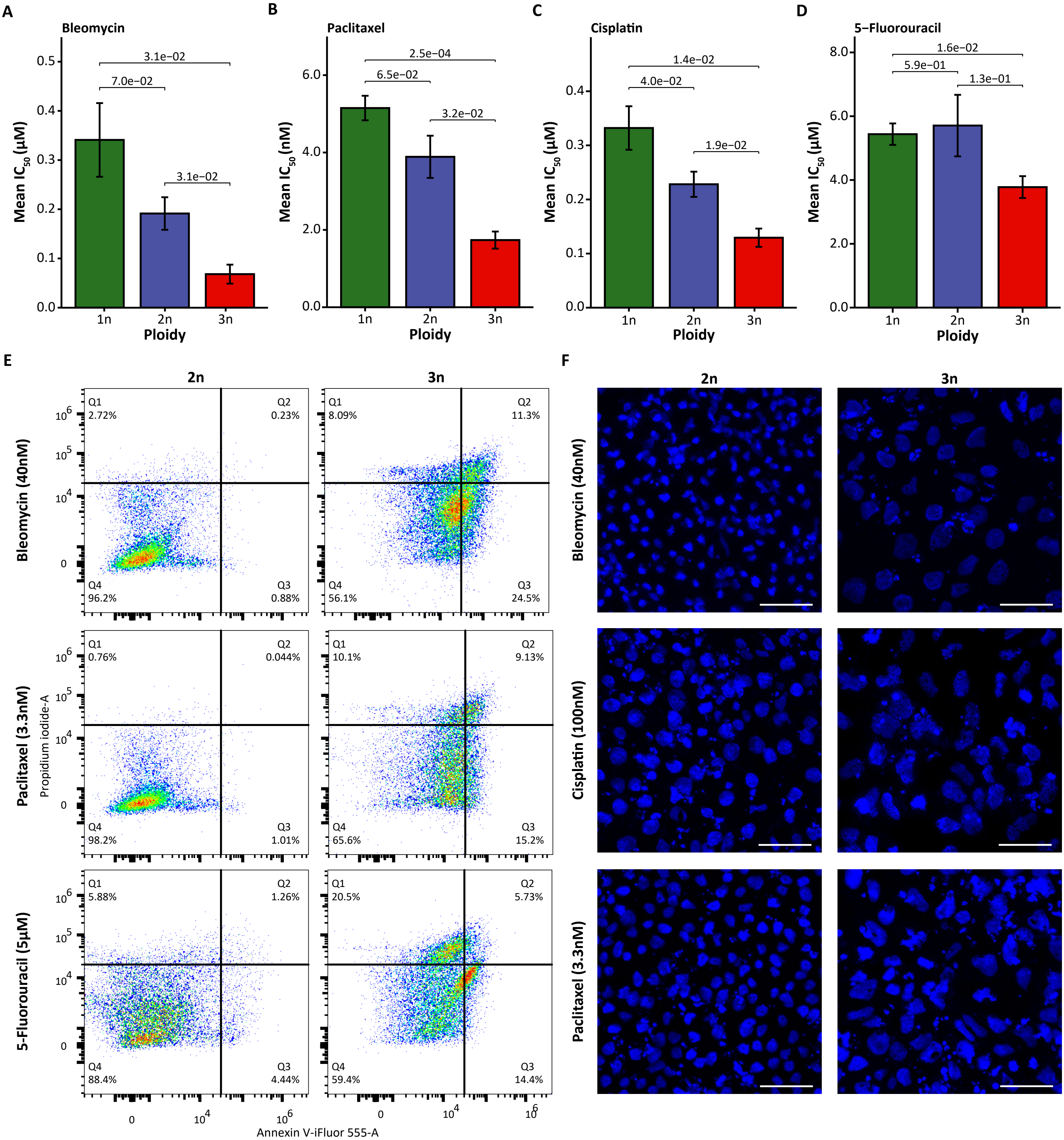

Supplementary Figure 1. Sensitivity assessment to anticancer drug exposure of hESCs with different ploidies. A-D) Mean IC50 values, calculated from the CellTiter-Glo® 2.0 intensity levels of the timepoint that showed the most difference in cell viability after exposure to different concentrations of bleomycin (A), paclitaxel (B), cisplatin (C) and 5-FU (D). The comparison was done by performing a student's *t*-test, with FDR-corrected p-values. Error bars represent the SEM. **E)** Representative flow-cytometry plots of diploid (left) and triploid (right) hESCs treated with bleomycin (40nM, top), paclitaxel (3.3nM, middle) and 5-FU (5µM, bottom) after apoptosis assay was performed. The analysis was done using the spectral Cytek Aurora flow cytometer. **F)** Representative images of diploid (left) and triploid (right) hESCs treated with bleomycin (40nM, top), cisplatin (100nM, bottom) and paclitaxel (3.3nM, middle) for 72 hours and then stained with DAPI. Scale bar = 50 µm.

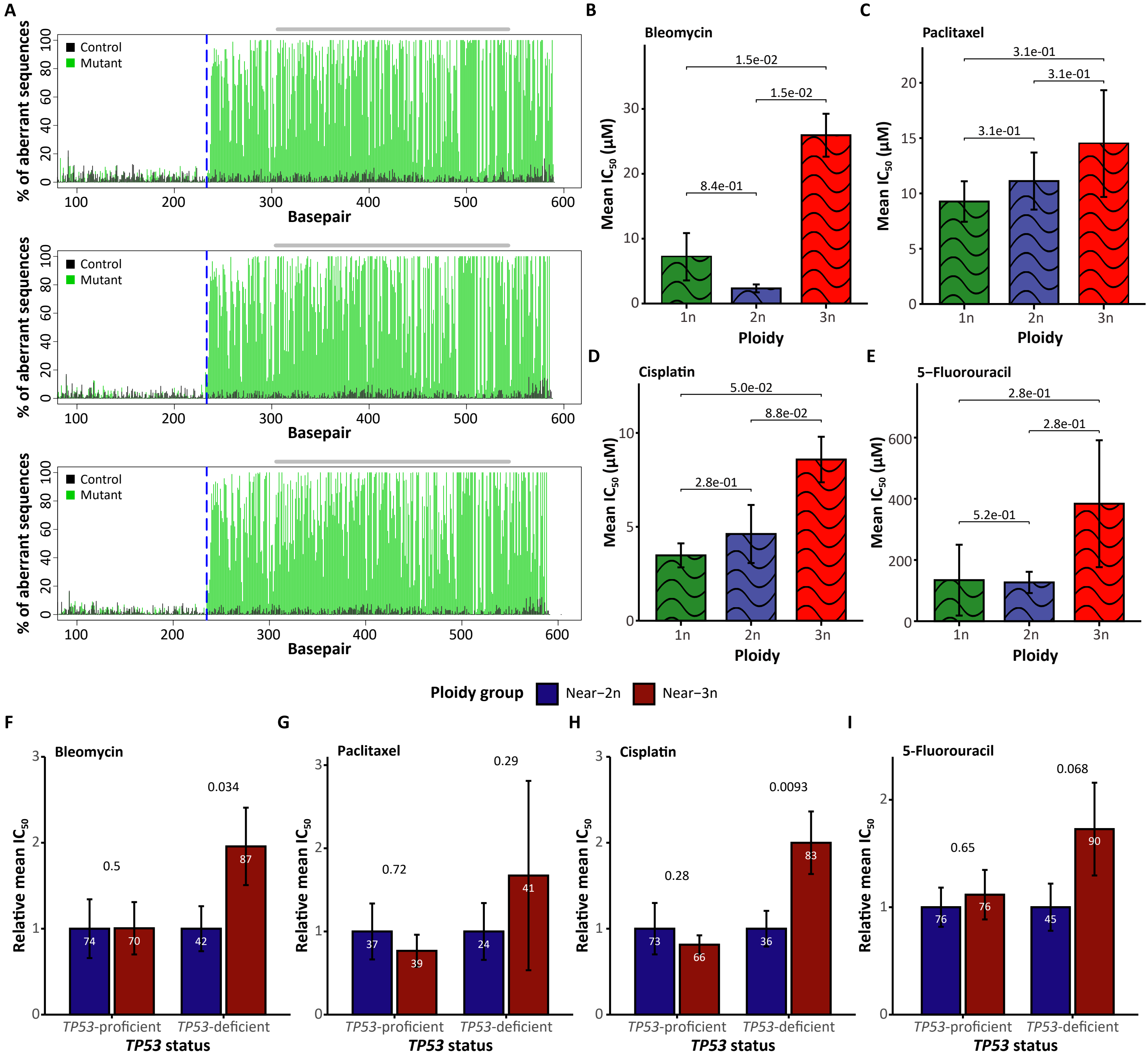

Supplementary Figure 2. Validation of CRISPR-Cas9 efficacy targeting *TP53* and chemotherapy sensitivity assessment of *TP53*-KO in hESCs and CCLs with different ploidy levels. A) Representative assessments of *TP53* gene editing efficiency of haploid (top), diploid (middle) and triploid (bottom) hESCs by decomposition of the Sanger sequencing results of each cell line using TIDE. The grey line indicates the region for decomposition and the blue dashed line indicates the estimated editing site. For each cell line, the percentage of aberrant sequences of WT cells is shown in black and the percentage of aberrant sequences of the same line that was mutated in *TP53* is shown in green (*TP53*-KO cells). **D-G)** Mean IC_{50} values, calculated from the CellTiter-Glo® 2.0 intensity levels of the timepoint that showed the most difference in *TP53*-null cells' viability after exposure to different concentrations of bleomycin (B), paclitaxel (C), cisplatin (D) and 5-FU (E). The comparison was done by performing a student's *t*-test, with FDR-corrected p-values. Error bars represent SEM. **F-I)** Sensitivity assessment by a comparison of the relative mean IC_{50} values obtained from the GDSC of near-2n and near-3n CCLs with a functional p53 (*TP53*-proficeint) or with a full *TP53*-LoF (*TP53*-deficeint) exposed to bleomycin (F), paclitaxel (G), cisplatin (H) and 5-Fluorouracil (I). The comparison was done by performing a one-sided student's *t*-test. Error bars represent SEM. Number of CCLs (n) compared in each ploidy group and *TP53* status is shown inside the bars for every treatment.

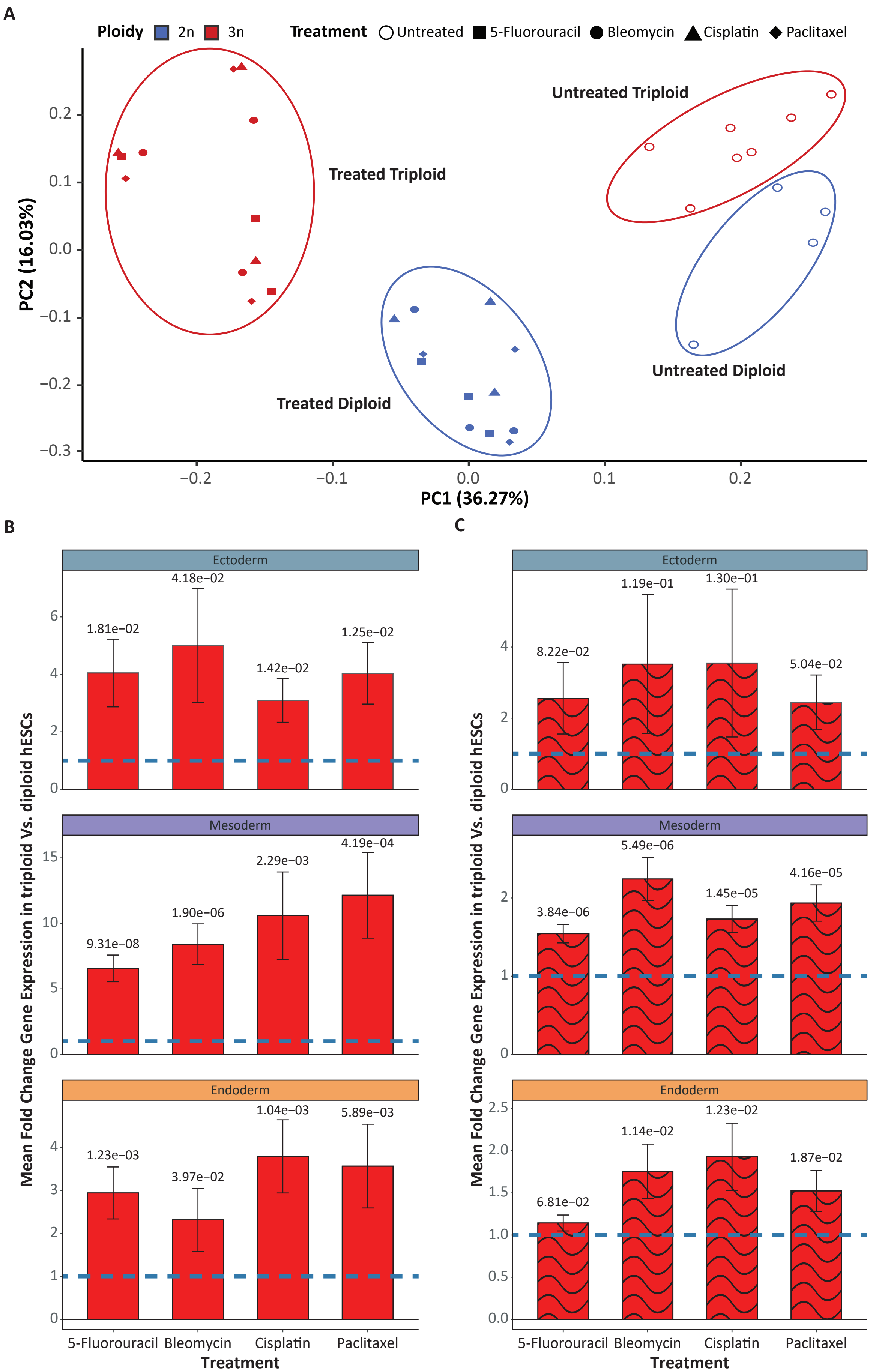

Supplementary Figure 3. Separation of treated and untreated hESCs based on gene expression. A) PCA plot of diploid and triploid hESCs based on whole genome trimmed mean of M values (TMM) expression levels. Cells were either untreated or treated for 48 with bleomycin (20nM), paclitaxel (3.3nM), cisplatin (100nM) and 5-FU (2.5µM) before sequencing. **B-C)** Mean relative gene expression levels (fold change) of specific germ layers marker genes in either WT (B) or *TP53*-KO (C) triploid hESCs compared to their diploid counterparts, treated with different chemotherapies. The diploid cells' relative gene expression level is represented by the blue dashed line in each plot. For each treatement and ploidy level, for both WT and *TP53*-KO hESCs, n = 3. p-values were calculated by a one-sided student's *t*-test. Error bars represent SEM.

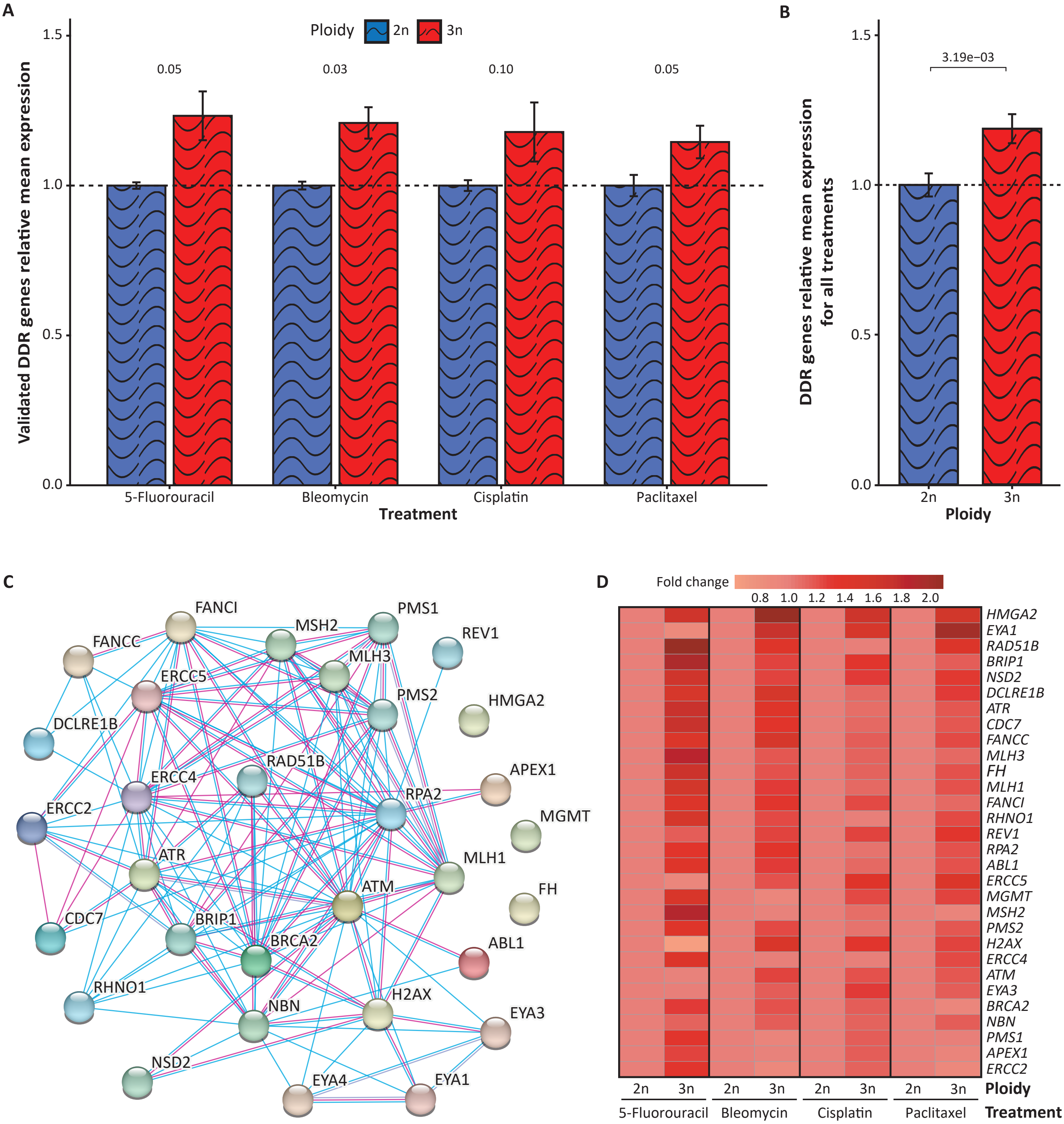

Supplementary Figure 4. DNA damage response of *TP53*-KO hESCs upon exposure to anticancer drugs. A) Mean relative expression levels of genes that were found to be directly linked to DNA damage repair in *TP53*-KO triploid hESCs compared to diploid cells exposed to the different chemotherapies. n=3. **B)** Overall relative expression levels of genes directly linked to DNA damage repair that were found to be highly enriched in *TP53*-KO triploid hESCs compared to diploid cells across the different treatments. n=12. **C)** STRING analysis of the genes directly linked to DNA repair. **D)** Heatmap displaying the mean relative expression levels (fold change) of the genes directly linked to DNA repair in *TP53*-KO triploid hESCs compared to diploid cells exposed to the different chemotherapies. n=3. The comparisons in (A) and (B) were performed by a one-sided student's *t*-test. Error bars represent SEM. All analyses were based on the expression levels of the genes listed in (C) and (D).
